# Bouncy Idea or Solid Practice? Exploring Industry Barriers in the Incorporation of Devulcanized Rubber into Compounds for Rubber Products

**DOI:** 10.3390/polym17111570

**Published:** 2025-06-05

**Authors:** Eric Roetman, Jelle Joustra, Geert Heideman, Ruud Balkenende

**Affiliations:** 1 Faculty of Industrial Design Engineering, Delft University of Technology, 2628 CE Delft, The Netherlands; j.j.joustra@tudelft.nl (J.J.); a.r.balkenende@tudelft.nl (R.B.); 2 Polymer Engineering, University of Applied Sciences Windesheim, 8017 CA Zwolle, The Netherlands; g.heideman@windesheim.nl

**Keywords:** circular economy, rubber, recycling, devulcanization, barriers

## Abstract

Devulcanization has the potential to help meet circular economy goals by recovering end-of-life rubber. However, the adoption of devulcanized rubber by manufacturers remains low at the industry level. Devulcanization value chains are complex and involve multiple stakeholders, including waste collectors, sorters, recyclers, compounders, manufacturers and regulatory bodies. This study investigated the barriers compounders and manufacturers face when incorporating devulcanized rubber into new compounds and identified primary underlying causes. The research was conducted through in-depth interviews with compounders and manufacturers of tires and general rubber goods, focusing on the technical, market, institutional, and cultural factors related to incorporating recycled materials, specifically devulcanized rubber. From the results, we identified a number of barriers faced by the industry. A key barrier was the heterogeneity of devulcanized rubber, which made it more difficult to add to new rubber compounds with consistent quality. Other barriers included a lack of standardization and coordination, along with misaligned regulations that hamper the market adoption of devulcanized rubber. This implies that increasing the uptake of devulcanized rubber at the industry level will not be achieved through technological advancements alone or isolated market interventions; instead, it requires comprehensive, systemic solutions.

## 1. Introduction

Although devulcanization techniques for rubber have been claimed for decades [1], recent years have seen significant technological advancements, making it increasingly suitable for rubber recycling and raising the potential to meet the goals of the circular economy [2,3]. Despite these technological advancements and considerable investments, the market uptake of devulcanized rubber remains low compared to other rubber recycling routes [2,4].

Devulcanization is an interesting technology that can be used to meet circular economy goals. During rubber goods production, rubber is made firm, elastic, and durable by vulcanization. In that process, sulphur cross-links are created on a molecular level between the rubber polymer main chains. Vulcanization enhances rubber’s durability and heat resistance but creates significant recycling challenges. Devulcanization is a group of recycling technologies with the objective to reverse vulcanization by selectively breaking the sulphur cross-links. Devulcanization processes aim to recycle rubber while retaining most of its original properties [3,5,6]. Devulcanized rubber can be added to new rubber during the compounding stage when raw rubbers are compounded with various additives and fillers to achieve specific properties [6,7,8]. A rough estimation of the total current European production capacity for devulcanized rubber is 100 kton/year (In Roetman et al. [4], 17 companies were identified in Europe that devulcanized rubber. The interviewed companies had an average capacity of 1.45 ton/h. The assumption is that these companies can run 4000 h on a yearly basis, leading to a total annual capacity of 100 kton of devulcanized rubber) [4].

Devulcanization is a unique approach to rubber recycling because it specifically aims to preserve the original properties of the recycled rubber [2,9]. For comparison, an overview of the main recycling methods is presented in Appendix A. Devulcanization is unique because of its aim to preserve the material properties, as opposed to other methods that often lead to degradation or deterioration of the rubber. Devulcanization is important because of the significance of rubber for the circular economy. Synthetic rubber is primarily made from fossil fuels through energy-intensive methods [10,11]. Natural rubber is a critical and potentially scarce raw material and its regeneration presents challenges [12]. Thus, devulcanization contributes to a circular economy by offering a solution by preserving both material groups. Further, it fits the circular economy goal of maintaining performance and value as much as possible [13].

Even though devulcanized rubber can be used to replace primary, virgin rubber, its composition is different. Devulcanized rubber contains raw, natural or synthetic rubber as well as the additives of the original compounds [2]. These additives include processing aids, vulcanization agents and reinforcing fillers [14,15]. Additionally, the performance and quality of devulcanized rubber depends on the devulcanization technology, as well as the properties, quality, and consistency of the rubber waste collected [5,6,16].

Despite its potential to at least partially replace virgin rubber compounds and thus reduce the carbon footprint [2], there is a low uptake of this material by manufacturers and compounders who could potentially add devulcanized rubber to their compounds. Their perspective is important to investigate, as a study on companies that devulcanize rubber in Europe identified the low uptake of devulcanized rubber as a significant barrier for their business case [4].

Rubber compounders and manufacturers perform a crucial role in the rubber value chain by developing formulations and blending rubbers with additives to meet specific requirements [15]. Rubber compounders are specialized companies that develop rubber formulations and blend rubbers with additives to achieve the desired properties. While some rubber manufacturers have their own compounding divisions, their primary goal is production of goods containing rubber. We distinguish two types of manufacturers: tire manufacturers and manufacturers of rubber goods. Tire manufacturers consume the majority of the global rubber supply. Table 1 shows the consumption of different types of rubber on a global level and Table 2 compares the consumption of general rubber goods and tire production in Europe. Table 3 presents import and export data for rubber products within Europe, highlighting the significance of rubber production in the region.

General rubber goods manufacturers produce finished or semi-finished rubber products, such as conveyor belts, shoe soles, waterproof materials, electrical insulation, adhesives, O-rings, seals, hoses and belts, and industrial gloves for various industries [15,17]. In these goods general-purpose rubbers, such as SBR and natural rubber, as well as specialty rubbers are used that meet specific industry requirements (e.g., fluorocarbon (FPM), which is resistant chemical and solvents) [18]. The rubber compounders and manufacturers are in the position to add devulcanized rubber to a wide range of compounds.

**Table 1:** Global rubber consumption in 2020 [19,20].

	Natural Rubber		Synthetic Rubber	
		Million Metric Tons		Million Metric Tons
Region	%		**%**	
Europe	8%	1.02	14%	1.99
China	43%	5.46	31%	4.40
India	8%	1.02	4%	0.57
Japan	5%	0.64	5%	0.71
USA	6%	0.76	11%	1.56
Rest of the world	30%	3.81	35%	4.97
Total	100%	12.7	100%	14.2

**Table 2:** Rubber consumption by industry in the EU and UK in 2020 [19,20].

	Natural Rubber		Synthetic Rubber	
		Million Metric Tons		Million Metric Tons
	%		%	
General rubber goods	27%	0.25	55%	1.09
Tires	73%	0.77	45%	0.90
Total	100%	1.02	100%	1.99

**Table 3:** Economic value of rubber products imported and exported from the EU in 2020 (excl. UK) [21].

	Imported	Exported
	€ Billion	€ Billion
General rubber goods	5.6	5.2
Tires	5.8	5.6
Total	11.4	10.8

In the academic literature, the main barriers identified for manufacturing to come to a circular economy are high startup costs, complex supply chains, challenging collaboration amongst companies, compromises on quality, and the high costs of disassembling products [22]. However, the specific reasons for the low uptake of devulcanized rubber have not been extensively investigated. Therefore, the main research question that will be addressed in this article is: What barriers do compounders and manufacturers encounter when incorporating devulcanized rubber into new rubber compounds, and what are the major underlying factors that contribute to these barriers? The studied factors include technical, market, institutional, and cultural factors, which are recognized in the academic literature as key to the circular economy transition [23].

The methods section outlines the selection of respondents, the type of interviews, and interpretation of these interviews. The results section presents key findings from the interviews. This section addresses the main subjects for the technical, economic, institutional, and cultural factors, while the discussion section interprets these findings by reflecting on potential interventions. Finally, the conclusion section summarizes the overall insights regarding the opportunities and barriers for devulcanization as a means to transition to a circular economy for the rubber industry.

## 2. Methods

For this study, a qualitative research method was used to identify the barriers for the uptake of devulcanized rubber and their underlying factors. We identified these barriers by interviewing representatives of compounding and manufacturing industries, the potential users of devulcanized rubber. We used purposive sampling [24], deliberately selecting organizations rather than randomly choosing them, and conducted in-depth interviews to identify key topics and gain an understanding of the underlying complexity [25,26].

Representatives for the interviews were selected from companies with in-depth knowledge and ample experience related to the addition of recycled materials to new compounds. The interviews were conducted in-person or via video conferencing, recorded, and transcribed for an accurate representation. The interviews were then coded and interpreted according to the coding scheme in Appendix C.

### 2.1. Selecting Respondents

This study focused on both compounders and manufacturers with experience of incorporating devulcanized rubber. We identified and approached potential participants through organizations with extensive networks in the rubber industry and through national sector specific associations for the rubber and tire industry in Europe. Companies based in Europe or with European subsidiaries were prioritized, although many were part of international corporations or holding companies. This generated an international view on the topic with an emphasis on Europe. We approached both tire manufacturers as well as general rubber goods manufacturers. Amongst the tire manufacturers, respondents were specifically selected from the 15 companies with the highest global turnover in 2023 [27].

Eight respondents were interviewed. Seven of these respondents had experience working with reclaimed and/or devulcanized rubber. Three of these respondents did not add devulcanized rubber to compounds for commercial products at the time of the interview but continued to do tests with new types of devulcanized rubber. One respondent, a compounder, had stopped adding devulcanized material because it could not be used as a drop-in, a direct replacement of virgin rubber. One respondent did not have any experience of working with devulcanized rubber but did have experience of working with non-vulcanized production waste. This allowed him to draw comparisons between the two materials, providing valuable insights and context for positioning devulcanized rubber in the analysis.

All interviewed companies were operating internationally, and they provided information not only about the European context but frequently also compared this context to the situation in other continents. Table 4 provides general information about the respondents and their companies.

### 2.2. Interview Topics and Coding Scheme

The interviews lasted about one hour. Prior to the interview, the respondents received an opening statement, including the interview topics (Appendix B). The interview topics were derived from the literature on barriers to the circular economy transition. A common approach in these research papers is grouping the identified topics into sets of factors: technological, market, institutional, and cultural. This emphasizes the need for a multidisciplinary framework to identify the barriers [23,28,29,30]. This framework was also applied to plastics recycling in supply chains in other studies [31,32,33]. Table 5 provides an overview of the factors and research themes identified from the literature.

### 2.3. Data Analysis

The interviews were coded using structural coding, an approach for analysing large segments of qualitative data along broad categories. This creates an overall framework that can be applied for in-depth analysis of these topics [41]. After the interviews, coding was done using QDA Minor. Supplemental codes were added to capture additional topics that emerged during the interviews. The interviews were interpreted and described along these codes. The coding scheme was adapted after analysing the interviews, leading to the grouping of some codes and the addition of others. The final coding scheme was based on the topics in Table 5 and can be found in Appendix C.

### 2.4. Comparison of Interview Data with the Literature

The literature on the use of devulcanized rubber by the industry is limited. Therefore, two recycled materials were selected from the academic literature for comparison, to gain a more in-depth understanding of the identified barriers and the extent to which these barriers are uniquely related to this type of recycling. This approach was used to determine if the identified barriers are found more often and to identify interventions that could facilitate the uptake of devulcanized rubber. Two materials were selected: recovered carbon black and mechanically recycled plastic.

Recovered carbon black was chosen as it is a recycled material with the potential to be added to rubber compounds in large quantities. It is retrieved from end-of-life rubber through pyrolysis and can be blended into new rubber compounds as a replacement for carbon black [42]. However, recovered carbon black has lower quality than the original material. One of the reasons is that its surface is often contaminated [43,44]. In a consultancy report, 12 companies were listed that in total recovered 20 kton/year of recovered carbon black in Europe, mainly in pilot factories. These companies announced plans to increase their production to 232 kton/year in the coming years [45]. Although the production capacity is currently relatively small, similar to devulcanization, it is expected to grow significantly in the coming years.

The second material selected was recycled granulate from the mechanical recycling of end-of-life thermoplastic waste [46]. This represents the bulk source of recycled polymer, accounting for more than 400 million metric tons in 2023 worldwide [47]. It is produced from a mixed range of input materials and is downgraded compared to the virgin raw materials.

By entering combinations of the main keywords of the identified research themes of Table 5 into Google Scholar and ScienceDirect and using terms such as ‘recovered carbon black’, the literature on these two groups of materials was identified. Only articles published after 2020 were included to obtain recent research findings on encountered barriers and interventions. The first 40 results were reviewed for each combination of keywords. If no academic literature was identified about recovered carbon black or thermoplastic recyclate, information was sought from white papers and consultancy reports. If information from such non-academic sources was used, this is explicitly stated in the text.

## 3. Results

The findings are presented according to the four categories outlined in the methods section: technical, market, institutional, and cultural factors. In each of these categories, the factors are described along with the specific results that emerged in the interviews. The results are provided in Table 6, Table 7, Table 8 and Table 9, which also show which respondents referred to particular topics by differentiating between compounders (C), manufacturers of general rubber goods (G), and tire manufacturers (T). In the next column, the identified references to comparable barriers found in the literature on recycled carbon black (rCB) and thermoplastic recycled granulate (TRG) are listed.

### 3.1. Technical Factors

Table 6 lists the barriers that were found related to the technical factors. The introduction of a devulcanized rubber as a new ingredient impacts the overall composition of rubber compounds. All six respondents that used devulcanized rubber first did additional research and development and then reformulated their rubber compounds. If the quality of devulcanized rubber was not fully consistent, even more research was needed.

**Table 6:** Barriers related to the technical factors.

*Barriers identified in interviews with compounders (C), general rubber goods (G), and tire manufacturers (T)*	***Respondents***			*Similar barriers identified in the literature about recovered carbon black (rCB) and thermoplastic recycled granulate (TRG)*
	*C*	*G*	*T*	
*Research theme:* overall quality of supplied recycled ingredients.				
**Not a drop-in replacement:** devulcanized rubber is not considered to be a drop-in replacement for virgin rubber, as the properties are not similar to virgin materials.	*2*	*2*	*2*	**rCB:** the scientific literature only mentions different properties for rCB [44,48]. A white paper: [49].
				**TRG:** [31,50,51,52].
*Research theme*: consistency in both the quality and quantity of the supply of recycled materials.				
**Heterogeneity:** respondents developed their own, exclusive compounds for unique products, which causes heterogeneous waste streams.	*2*	*2*	*2*	Causes of heterogeneity: **rCB:** [43,53].
				**TRG:** [54].
**Reduced material properties:** devulcanized rubber was derived from mixed sources, which resulted in a heterogeneous mixture with inconsistent quality.	*3*	*1*	*1*	**rCB:** [44,55].
				**TRG:** [32,34,46,51,56,57].
**Unintended side effects:** the addition of recycled material could lead to unexpected side effects during processing or use.	*2*	*1*	*1*	**rCB:** [44,58].
				**TRG:** [57,59,60].
*Research theme*: Required changes in compound formulations and product designs to use recycled ingredients.				
**Need to reformulate compounds:** compounds needed to be reformulated to compensate for the composition of devulcanized rubber.	*2*	*1*	*2*	**rCB:** [44,58].
				**TRG:** [61,62].
**Numerous formulation variations:** general rubber goods manufacturers and compounders often had dozens of different compound formulations for specific products, all of which needed to be adapted.	*3*	*1*	*-*	**rCB:** No literature identified. However, the literature does mention the limitations of rCB for specific purposes [44,48]
				**TRG:** No literature identified but limitations of TR were mentioned for specific purposes, such as applications in the automotive industry [56,63].

In recent years, more types of devulcanized rubber have entered the market, mainly sourced from different types of vulcanized production waste and rubber recovered from end-of-life tires. Respondents who tested these new types of devulcanized rubber mentioned that the technical properties were better than those of materials previously available. Despite these improvements, the devulcanized rubber was only added in small quantities for critical applications; respondent T2 mentioned that only 1–2 percent was added to the most critical compounds. Adding a larger amount to these compounds was deemed too risky.

The barriers, as experienced by the respondents, primarily arose from issues related to the inconsistent quality of devulcanized rubber, which was partially caused by the variability in feedstock from rubber waste streams. The negative effect of heterogeneously sourced material was also a challenge for recovered carbon black [44,48] and recycled granulate from thermoplastic waste [31,50,51]. Until recently, recovered carbon black was rarely used in the rubber industry due to inconsistent properties [64]. Recent studies have shown improved quality consistency by reducing the ash content [42,43,65]. As these methods are still under development, the recovered carbon black industry also focusses on negating any negative effects [66], as is currently the case for devulcanized rubber.

In the case of the mechanical recycling of thermoplastic waste, the composition was made more homogeneous by automated separation and sorting [67] and with detection of the types of plastics by applying state-of-the-art vision technologies [68,69]. However, the quality was still inconsistent due to, e.g., layered or non-separable plastics and contaminations on the plastic [34,46,51,56]. An underlying reason was the municipal systems of plastic waste collection and separation before recycling, leading to additional contamination and mixing of materials [70]. This mirrors the challenges associated with car tires as a source of devulcanized rubber, as they consist of multiple layers of different rubber compounds and are gathered through diverse collection systems across Europe, each with its own processes and efficiencies [4].

### 3.2. Market Factors

Table 7 lists the barriers observed related to the market factors. Although devulcanized rubber had a lower purchasing price than virgin rubber, respondents did have to incur additional costs. These costs primarily arose from the effect of this new ingredient. Respondents noted that they had additional costs to compensate for the lower consistency in the quality of the material. More research and development were also needed to make the devulcanized rubber compatible with the rubber compound. Respondents C2 and G2 also included costs for troubleshooting for recycled materials in their calculations if unexpected effects occurred during processing or to address possible customer complaints. To account for these costs, respondent C2 mentioned that when new compounds needed to be developed for a customer, they required the customer to share in the associated development costs.

**Table 7:** Barriers related to the market factors.

*Barriers identified in interviews with compounders (C), general rubber goods (G), and tire manufacturers (T).*	***Devulcanization respondents***			*Similar barriers identified in the literature about recovered carbon black (rCB) and thermoplastic recycled granulate (TRG).*
	*C*	*G*	*T*	
*Research theme:* investment costs for compounding with recycled ingredients.				
**Additional expenditures:** additional expenditures to compensate for a less consistent quality of the material.	*2*	*2*	*2*	**rCB:** no literature identified.
				**TRG:** [31,33,35,51,56].
*Research theme:* costs of recycled ingredients versus virgin ingredients.				
**Low purchasing cost:** devulcanized rubber was cheaper than virgin rubber and less valuable on the market.	*1*	*1*	*2*	**rCB:** [71,72].
				**TRG:** [31,73].
*Research theme:* market dynamics for products with recycled ingredients.				
**More customer requests, greater diversity of supply:** in business-to-business sales, more customers were requesting rubber compounds that included biobased or recycled materials, and an increasing number of suppliers were offering recycled materials.	*2*	*2*	*2*	**rCB:** [72]. In additional to this source, the topic was raised by tire manufacturers in their white paper [74].
				**TRG:** the literature describes an imbalance between supply and demand in terms of availability, quality, and pricing [31,75].
**Increased obligations:** more requirements for recycled materials than for virgin materials; companies had to undergo additional audits and environmental impact assessment from their customers.	*3*	*1*	*3*	**rCB:** no literature identified.
				**TRG:** [29,33,76].

The market dynamics were also different from virgin materials. A reason was that respondents had to engage with new potential suppliers of recycled materials. For devulcanization, many of these suppliers were startups that needed launching customers. Another reason was that respondents T2, T3, C1, C2, and C3 noted more requests for rubber products that contained recycled or biobased materials. The sustainability goals set by the automotive industry were an important driver. Respondent T2 cited an example of a car manufacturer that requested a tire that contained at least 40% sustainable materials.

Respondents C2, C3, and G1 explicitly mentioned that when they supplied products with recycled materials to such customers, they were expected to collaborate on the sustainability reporting of their customers. They needed to provide additional data to their customers related to the customer sustainability goals, such as a reduced CO_2_ footprint. This included information about the recycled materials and environmental assessments.

According to the literature, market demand of recycled materials is vulnerable to fluctuations in the prices of the virgin materials, which is described for carbon black [71,77], plastics [31,73], and rubber [78]. In Europe, plastic recyclers went out of business due to the low prices of virgin plastics in 2024 [79]. In contrast, the business case for recovered carbon black through tire pyrolysis is not only dependent on carbon black but also on pyrolysis oil (naphtha), which can be blended into marine fuel and allows shipping operators to meet their obligations under the FuelEU Maritime Regulation [80]. Thus, although the market dynamics for these recycled materials differ, their prices are still influenced by factors such as supply and demand, production costs, and the prices of competing virgin materials.

### 3.3. Cultural Factors

Table 8 lists the barriers observed related to cultural factors. Respondents highlighted many of their efforts to increase the use of recycled materials in rubber compounds. However, respondents T1, T2, T3, C1, C2, C3, and G2 emphasized that product quality and overall costs were not allowed to be compromised. In contrast, G1 actively sought markets that accepted products made with recycled materials.

**Table 8:** Barriers related to the cultural factors.

*Barriers identified in interviews with compounders (C), general rubber goods (G), and tire manufacturers (T)*	*Devulcanization respondents*			*Similar barriers identified in the literature about recovered carbon black (rCB) and thermoplastic recycled granulate (TRG)*
	*C*	*G*	*T*	
*Research theme:* position of sustainability in company culture.				
**Limited room for compromises on quality and costs:** companies set clear sustainability goals, but have no willingness to compromise on either cost or quality.	*2*	*2*	*1*	**rCB:** no literature identified.
				**TRG:** [31,51,56,81].
*Research theme:* collaborative efforts to align and increase the use of recycled materials in new products.				
**No coordination:** coordinated industry wide efforts were identified to increase recovered carbon black but not devulcanized rubber.	*-*	*-*	*3*	**rCB:** no academic literature identified but other sources mention that tire manufacturers collaborated with rCB suppliers and other stakeholders [74].
				**TRG:** Although no academic literature was identified, several inter-company collaborations were recognized: [82,83].
*Research theme:* end user appreciation for increasing the uptake of recycled ingredients.				
**Low consumer demand for recycled materials:** no demand was identified for tires made from sustainable materials.	*2*	-	*2*	**rCB:** no literature identified.
				**TRG:** [31,33,84].

The demand for more recycled materials in rubber came from customers from the industry, not from consumers. According to respondents T2 and T3 the demand from end users was low and they did not spot any willingness among consumers to pay a premium for rubber products containing recycled material, such as tires. Respondent T2 mentioned that his company wanted to contribute to a campaign aimed at raising awareness among consumers to increase the acceptance of recycled and biobased materials in tires.

No industry-wide collaboration or coordination was mentioned by any of the respondents for an industry-wide commitment to the uptake of devulcanization. In contrast, respondents did mention such collaboration for recovered carbon black, which was also mentioned in consultancy reports and a white paper by the rubber industry [45,74]. For recycling of thermoplastic waste, several industry wide collaborations were identified that aimed to develop more recyclable products, coordinate waste collection systems and innovate recycling techniques [82,83,85].

### 3.4. Institutional Factors

Table 9 lists the barriers observed related to the institutional factors. Respondents T1, T2, and T3 considered quality standards to be important for the uptake of recycled materials. However, they identified such standards for recovered carbon black but not for devulcanized rubber. This is in line with the literature, in which standards were identified for recovered carbon black [55,86] and thermoplastic recycled granulate [87,88,89] but not for devulcanized rubber [3,4].

**Table 9:** Institutional factors.

*Barriers identified in interviews with compounders (C), general rubber goods (G), and tire manufacturers (T).*	*Devulcanization respondents*			*Similar barriers identified in the literature about recovered carbon black (rCB) and thermoplastic recycled granulate (TRG).*
	*C*	*G*	*T*	
*Research theme*: coordinated efforts for alignment and standardization to increase the uptake of recycled materials.				
**No specific standards:** respondents mentioned standards for other recycled materials but were not aware of such standards for devulcanization.	*-*	*-*	*3*	**rCB:** a specific ASTM standard, including a test method were developed for rCB [90,91].
				**TRG:** Standards were developed by industries for thermoplastic recyclate [76].
*Research theme*: accountability mechanisms of manufacturers that impact the use of recycled ingredients.				
**Liability concerns:** Companies were accountable for their products, including liability for any damages caused by those products or their components. This made the companies more risk-averse and reluctant to increase the amount of recycled materials.	*2*	*1*	*1*	**rCB:** no literature identified.
				**TRG:** no literature identified.
*Research theme*: alignment of regulations across sectors and geographical regions that impact the use of recycled ingredients.				
**National regulatory differences:** differences in interpretations of EU waste laws made it increasingly difficult to ship rubber across borders.	1	1	1	**rCB:** no literature identified.
				**TP:** [28,35].
**Regulations from other domains:** a key concern was meeting multiple regulations concerning environmental and safety goals.	1	1	3	**rCB:** no literature was identified
				**TRG:** [28,31,35,92,93].
**Need for alignment of regulations:** respondents indicated a need for better governmental alignment of regulations that directly or indirectly impact the adoption of recycled materials.	-	1	3	**rCB:** no literature was identified.
				**TRG:** [28,31,35,92].

Respondents T1, T2, and T3 noted that legislation could lead to an increase in the uptake of recycled materials in rubber but T1, T2, T3, C2, and G1 also expressed concerns about the implementation of upcoming legislation, such as a blending obligation of non-fossil ingredients in rubber. This was considered to have negative side effects by respondents T3, C1, and G1. The reasons were: effect on product quality, increased regulatory pressure, and its impact on pricing compared to international competition.

The current legislative framework in Europe was identified as a barrier to increase the uptake of recycled materials. A specific concern expressed by respondents T1, T2, and T3 was that they already had to meet multiple environmental and safety regulations. These could conflict with the aim to increase recycling rates. Respondents T1, T2, T3, and G3 also mentioned uncertainties related to the effect of upcoming and future considered regulations that could make it more difficult to rely on recycled materials. For this reason, respondent T1 mentioned that they not only conducted a technical check but also a legal check to guarantee that such materials could be used in the coming 10 years. A first example mentioned by T2, T3 and G1 was the EU Deforestation Regulations [94], which could imply that the origin of recycled natural rubber had to be demonstrated, which is challenging in an open-loop recycling system. A second example mentioned by respondents T2, T3, and G1 was that recycled rubber is now considered exempt from Registration, Evaluation, Authorisation, and Restriction of Chemicals (REACH) because the material has already been registered during the initial production. However, the European Chemicals Agency is considering that recycled materials, such as rubber, need to be demonstrably free of substances of concern [95]. This uncertainty of such upcoming regulation is also described in the literature about thermoplastic recycling [92,96].

Respondents T3 and G2 noted that European member states are allowed their own interpretation and implementation of EU legislation for waste and trade, which made it more difficult for their companies to work and ship across borders [97,98], directly leading to higher costs. For this reason, respondents T1, T2, T3, C2, C3, and G1 considered better alignment and more coherent implementation of regulations an important condition to increase recycling rates. As became evident from both the interviews and the literature in Table 9, the regulatory issues are a widely shared concern within the plastics and rubber industry.

## 4. Discussion

This study identified barriers across the technical, market, institutional, and cultural factors faced by compounders and manufacturers when incorporating devulcanized rubber into new rubber compounds, along with their underlying causes. Respondents from tire manufacturers, general rubber goods manufacturers, and compounders were interviewed as experts on processing recycled materials, with a specific focus on devulcanized rubber. The respondents reflected on their experiences with the addition of devulcanized rubber and other types of recycled materials to their compounds. In this discussion we reflect on these observations by focusing on how devulcanized rubber can potentially be incorporated in larger quantities into new compounds and identifying system interventions to achieve this goal.

A recurring topic among the technical factors is that devulcanized rubber has a heterogeneous composition, which is a clear barrier for the uptake of rubber. This heterogeneity is partially inherent to devulcanization, as a devulcanized rubber contains both the original rubber polymers as well as the additives that were used to create the new rubber compound [6]. This also arises from the heterogeneity of rubber waste sources. If such a large variation in rubber products enters waste systems, the devulcanization material becomes inconsistent, thus reducing quality [6,16]. Therefore certain companies devulcanize rubber from specific sources, such as truck tire tread rubber, to obtain devulcanized rubber with a more consistent quality [4,5,16]. This is an example of a closed-loop/closed-source system, implying that end-of-life materials from a well-controlled source are recycled at relatively high quality and value [99]. Other research showed that the closed-loop/closed-source concept is based on the premise that the original equipment manufacturers take responsibility for collection to arrive at a relatively homogeneous input stream from selected sources [100].

An important barrier among the market factors is that recycled materials are valued lower by compounders and manufacturers than new, virgin rubber. A reason is the required additional research and development costs, as well as potential risks related to product liability. An underlying reason is a lack of standardization for devulcanization, which could be improved by setting standards, as were identified for recovered carbon black [90] and thermoplastic recycled granulate [76]. Additionally, compounders could specialize in blending devulcanized rubber with virgin rubber compounds to create materials with devulcanized content and a more consistent quality for manufacturers. Companies that specialize in this type of blending were identified for recovered carbon black, such as [66], and thermoplastic recycled granulate, such as [101]. Thus, by standardizing and consolidating the devulcanization value chain, opportunities could arise to enhance quality and potentially increase the value of devulcanized rubber.

A key barrier among the cultural factors is the lack of industry-wide engagement in preserving the value and usability of rubber. Devulcanization research and development strongly focuses on technology development, hardly addressing other factors that limit implementation [4]. In addition to technological solutions for improved sorting and separating, companies could be creating distinct material recycling loops within their respective value chains, as identified in plastics recycling [102]. This requires an involvement from compounders, manufacturers, distributors, and recyclers and necessitates infrastructure and appropriate regulations to facilitate these closed-loop recycling systems [99]. Given the diverse range of rubber products and markets, multiple recycling chains can be established to create effective closed-loop/closed-source systems, each tailored to specific material streams where applicable.

A recurring institutional barrier is the absence of appropriate regulations. When looking at improvements, it is important to also take plans by the European Union into consideration. In the Circular Economy Action Plan for 2030 [103] a European blending obligation was announced, which mandates the inclusion of non-fossil ingredients (either biobased or recycled) in plastics and rubber. This obligation could boost the uptake of recycled materials in general, but the results of this study showed that compounders and manufacturers have a strong preference for materials that can be blended into compounds without having to make any modifications to their recipes. However, modifications are needed in compounds to compensate for the additives in devulcanized rubber [6]. For this reason, such an obligation may increase the uptake of other types of biobased and recycled materials, but not necessarily devulcanized rubber.

Another potential legislative intervention is extended producer responsibility (EPR), which can be effective for collecting waste and creating a secondary raw material market [104,105]. These systems are a collective responsibility of manufacturers but also importers, organized by producer responsibility organizations. Such organizations exist in several European countries and are aimed at the collecting of end-of-life tires [106]. This has not yet resulted in the most optimal material reprocessing from a circular economy perspective [106,107]. Due to the collective responsibility of all manufacturers and importers, ERP systems are geared towards open-loop recycling. This poses a particular obstacle for devulcanization, as a closed-loop recycling system is desirable to reduce the heterogeneity of devulcanized rubber. Thus, an ERP system, in which manufacturers carry a collective responsibility does not provide the incentive needed to stimulate rubber recycling by devulcanization.

The drawbacks associated with the blending obligations for non-fossil ingredients and EPR systems highlight the need for a more comprehensive approach to address the complexities, as outlined in this study. A system would be needed that focusses on creating both the infrastructure as well as industry collaboration to achieve the highest possible closed-loop recycling. To achieve this, policymakers should reconsider which stakeholders need to be involved to achieve the most optimal use and recycling of rubber. It is recommended as a topic for future research to study how an integrated approach could increase the circular economy for the rubber industry, specifically for devulcanization. This study demonstrates that achieving this goal requires more than just technological advancements; to reach higher quality standards on an industrial scale, collaboration across the supply chain is essential to enable closed-loop recycling via devulcanization.

The analytical framework depends on choices made during the analysis, which could restrict the scope of interpretations. Since most respondents were from Europe, the geographical validity of the results may be limited.

## 5. Conclusions

This study focused on identifying the barriers compounders and manufacturers face when using devulcanized rubber in new rubber compounds and finding the underlying causes to these barriers. It is demonstrated that significant changes are needed to increase the uptake of devulcanized rubber in the transition to a circular economy. It provides greater insight into the barriers, interconnections, and underlying causes, while also identifying opportunities for improved policy and collaboration, ultimately supporting the transition to the circular economy.

Most academic research on the topic of devulcanization has focused strongly on the technical aspects [2,3,108,109], but this study explored other factors influencing the uptake of devulcanized rubber that are essential to progress devulcanization beyond its niche in the industry. We investigated the technical, economic, institutional, and cultural factors that affect the uptake of devulcanized rubber as an ingredient in new rubber compounds.

Reflecting on the current situation, respondents identified barriers stemming from a variety of interrelated factors. Similar challenges have also been documented for recovered carbon black and thermoplastic recycled granulate, such as the lower homogeneity of recycled materials compared to new, virgin materials, leading to inconsistencies in quality. Barriers that were more specific to devulcanized rubber include limited industry collaboration and the need for greater standardization, both of which are important to improve efficiency and ensure consistency across the industry.

To produce high-quality devulcanized rubber, multiple interconnected barriers must be addressed. Overcoming these challenges on the industry level calls for systemic changes and effective collaboration among key stakeholders. Several approaches developed for other recycled materials could be worthwhile to increase the uptake of devulcanized rubber: industry standards, harmonized regulations, and closed-loop/closed-source recycling strategies.

## Figures and Tables

**Table 4:** Information about the respondents.

#	Type of Company	Role Within Company	Reference Code
1	Tire manufacturer	Research and Development Manager	T1
2	Tire manufacturer	Research and Development Manager	T2
3	Tire manufacturer	Corporate Affairs Manager	T3
4	Compounder	Director	C1
5	Compounder	Research and Development Manager	C2
6	Compounder	Research and Development Manager	C3
7	Producer of general rubber goods	Director	G1
8	Producer of general rubber goods	Production Manager	G2

**Table 5:** Interview topics.

**Technical factors** are the material-related aspects and the industrial activities to incorporate recycled ingredients into compounds for new rubber.		
**Identified research themes**	**Sources**	**Key words**
Overall quality of supplied recycled materials.	[31,33,34,35,36]	Quality
Required changes in compound formulations and product designs to use recycled materials.	[31,36,37]	Compound formulation, product design
Consistency in both the quality and quantity of the supply of recycled materials.	[33,38]	Consistency, quality, supply
**Market Factors** are the aspects related to the cost, pricing and market acceptance.		
Identified research themes:	Sources	
Investment costs for compounding with recycled materials.	[31,33]	Investments, costs
Costs and pricing of recycled materials versus virgin materials.	[31,32,33,35]	Cost, pricing, recycled, virgin
Market dynamics for products with recycled materials.	[32,33,39]	Market dynamics
**Cultural factors** are related to how a culture is collaborative and focused on engaging in the circular economy, as well as aligning value to facilitate the use of recycled materials.		
Identified research themes:	Sources	
Position of sustainability in company culture.	[31,33,39]	Sustainability, company/organisation, culture
Coordinated efforts to align and increase the use of recycled materials in new products.	[33,35,40]	Coordination, alignment
End user appreciation of increasing the uptake of recycled materials.	[32,33]	End user, customer, appreciation
**Institutional factors** are the policies, rules, regulations, taxes and infrastructure that directly or indirectly influence the decision-making of companies.		
Identified research themes:	Sources	
Efforts towards industry alignment and standardization to increase the uptake of recycled materials.	[31,33]	Alignment, standards
Accountability mechanisms of manufacturers and compounders that impact the use of recycled materials.	[33,35,39]	Accountability/ responsibility, mechanisms
Alignment of regulations across sectors and geographical regions that impact the use of recycled materials.	[31,35]	Alignment, regulations, sectors, regions

## Data Availability

The data utilized in this study cannot be made publicly available as it contains proprietary company-specific information.

## Appendix A. End-of-Life Methods for Rubber

The following methods were identified in the literature [2,110].

	**Feedstock**	**Working Principle**	**Applications**
Devulcanization	Rubber crumbs or powder	Devulcanized rubber compounds are aimed at a higher quality than reclaimed rubber. Devulcanization is a process that aims to selectively break the cross-links while keeping the rubber polymers intact. This is achieved through various methods, such as exposing the rubber to heat, chemicals, and shear strength. The result is a rubber compound that can be re-vulcanized [3,9,110].	Devulcanized rubber can be used to replace virgin rubber and additives. In most cases, this material is blended into new rubber compounds to compromise for any loss of quality [5,6]. Additionally, devulcanized rubber is added to bitumen to partially replace the plasticizer SBS [4,111].
Reclaimed rubber	Rubber crumbs or powder	On the molecular scale, in a reclaim process, both the rubber polymers and cross-links and are randomly broken. The input of an industrial reclaim process consists of rubber crumbs or powder, which are converted into a rubber compound that can be re-vulcanized [3,9].	Reclaimed rubber is mostly added to rubber compounds and then re-vulcanized. It is mostly used for less critical applications or added in small quantities to new rubber compounds [9].
Binder systems	Rubber crumbs or powder	For certain applications, the granulated rubber is bound with a polyurethane or a thermoplast [112,113]. Additionally, the surface of the rubber powder can be treated for better bonding in a rubber compound [114].	This material can be used for various products, such as rubber tiles and mats and underlayment for floors [113].
Pyrolysis	Rubber crumbs or powder	Pyrolysis is a thermal process in which rubber compounds are decomposed by heat (400–800 degrees Celsius) in the absence of oxygen into smaller molecules, which leads to oil, carbon black, and gas [110].	Pyrolysis oil can be used as a fuel and in theory for producing new monomers to create new plastics and rubber [115]. The carbon black can replace certain grades of virgin carbon black [116]. Lastly, the gas can be a source for energy [117].
Retreading	Passenger car tires, tuck tires, airplane tires, and off-the-road tires	In retreading the tread of a worn-out tire is removed. A new rubber tread compound is vulcanized onto the tire. Retreading is different from the recycling process, because the carcass of a tire can be reused [118]. The removed rubber from the tread can be recycled by another method, such as devulcanization [4]	Retreading is primarily used for tires from planes, truck, agricultural tires and sometimes passenger car tires [119].
Mechanical grinding	Different types of tires and rubber products, such as conveyor belts	Rubber is sorted from steel, canvas and other contaminants and is then mechanically processed into crumbs or powder of various sizes [113]. An alternative to mechanical processing is cryogenic shattering [120].	Rubber crumb or powder is used as a feedstock for various recycling methods, such as reclaim and devulcanization [9]. Additionally, rubber crumbs can be utilized as artificial turf on sports fields or recreational surfaces [120]. Rubber powder is also used as a simple filler material that can be added in small quantities to new rubber compounds [114]. Rubber crumbs are used as an additive for tarmac/asphalt [113].
Incineration	Tires and other end-of-life rubber products	Tires are disposed of by incineration, but this process produces toxic emissions [110]. Rubber is incinerated in cement plants [2] as an alternative source of heat, which produces less toxic emissions than coal [121]	Incineration can be used to recover 32.6 MJ/kg of embedded energy in tires [110].
Landfill	Tires and other end-of-life rubber products	Globally, 55 percent of all tires are dumped in landfills [122]	Large quantities of landfilled tires are known to pose a significant fire hazard. Additionally, they are toxic to soils and groundwater, and when burned, they cause air pollution [123]

## Appendix B. Opening Statement and Topic List Used for Interviews

### Appendix B.1. Opening Statement

My name is Eric Roetman, and I am a researcher at Windesheim University of Applied Sciences and a PhD candidate at TU Delft. I am conducting interviews with representatives from European companies that have devulcanized tires. I would like to interview you as a representative of one of these companies. During the interview, I aim to gather information about the key opportunities and barriers of adding recycled rubber to new rubber compounds.

During the interview, I will ask a set of questions on this topic. By posing these questions to you and representatives of other companies, we hope to gain an overview of the opportunities and barriers. The information will be recorded using a voice recorder and then transcribed. You will receive a copy of the main conclusions and have the opportunity to respond, suggest changes, or address any misinterpretations. If we wish to use non-anonymized quotes, or quotes traceable back to you, from the interview, we will contact you and only proceed with your explicit approval.

The aim of these interviews is to collect data for publication in an academic article and a PhD thesis. The information collected will be handled with great care, and the outcomes will be published in a manner that prevents identification of you or your company. Information traceable back to you will not be shared beyond the research team. The information from the interviews will be stored in a data repository by Hogeschool Windesheim (University of Applied Sciences).

Your answers in this study will be kept confidential to the best of our ability. Your participation in this study is entirely voluntary, and you can withdraw at any time.

If you have any questions or concerns about the interview or handling of the data, you can contact me directly by sending an email to e.c.roetman@tudelft.nl or calling me at [phone number].

### Appendix B.2. Interview Topics

General

1. Why did you start to incorporate recycled materials in rubber products/compounds?
2. What type of recycled materials?
3. Since when do you incorporate recycled rubber into your products/compounds? Why at that moment?
4. Have you applied recycled material to a new type of product or to existing products?
5. What new skills, facilities and resources did you need in your company to add recycled materials to rubber?
6. Up to what degree do you experience an advantages or disadvantages of being one of the first companies that incorporates recycled material in rubber?

Technical factors

1. What types of recycled source material (devulcanized, reclaimed, micronized rubber material, etc.) are most and least suitable for you to add to your rubber compounds? How does devulcanization fit in this list?
2. What product categories are most suitable products to incorporate such recycled materials?
3. How do you experience the performance and processability of recycled source material compared to virgin rubber material?
4. To what degree do the properties of your recycled source material pose challenges, and how can you mitigate these challenges?
5. How would you rate the availability of the recycled source materials for production?
6. How do you assess the quality of the supplied rubber material, specifically regarding contamination such as the presence of PAHs?
7. Did you have to change your production process to process recycled rubber, and why?
8. Up to what extent did you have to change your research and development process for processing recycled rubber?

Market factors

1. How would you evaluate the overall reliability of suppliers of recycled source material?
2. Up to what extent is it an added value for your customers that you include recycled content in products, or do they have reservations?
3. Up to what extent did encounter additional costs when incorporating recycled materials into your products? Includes upfront costs?

Institutional factors

1. Did upcoming regulations positively or negatively influence your decision to incorporate recycled rubber into your products?
2. Up to what extent have you benefitted from financial incentives (e.g., subsidies) that encouraged you to use recycled materials in manufacturing processes?
3. (How) did industry standards positively or negatively influence your decision to incorporate recycled rubber into your products?

Cultural factors

1. Up to what degree do you perceive a willingness amongst customers to add recycled materials?
2. Up to what extent is your engagement with your suppliers of recycled material different from suppliers of virgin materials?
3. How does the addition of recycled material to products affect your relationship with your compounder(s)?

Closing the interview

1. Is there any topic that is not addressed during this interview and you would like to address?

## Appendix C. Interview Coding

**Technical factors** are the material-related aspects and the industrial activities to process recycled ingredients into compounds for new rubber.
**Identified research themes**
Overall quality of supplied recycled materials - Performance devulcanized material - Performances (other) recycled materials - Technical challenges recycled materials - Suitability recycled materials for rubber products
Required changes in compound formulations and product designs to use recycled materials - Required changes to rubber compounds - Additional research and development - Required changes to production processes
Consistency in both the quality and quantity of the supply of recycled materials - Availability of recycled materials - Quality of recycled materials
**Market factors** are the aspects related to the cost, pricing, and market acceptance.
Identified research themes:
Investment costs for compounding with recycled materials - Required skills, facilities, resources - Additional costs to include recycled materials
Costs and benefits of recycled materials versus virgin materials - Additional costs of adding recycled materials - Financial benefits to include recycled materials
Market dynamics for products with recycled materials - Availability suppliers and supplies - Reliability of suppliers - Effect of adding recycled materials on trade
**Cultural factors** are related to the extent to which a culture is collaborative and focused on engaging in the circular economy, as well as aligning value to facilitate the use of recycled materials.
Identified research themes:
Position of sustainability in company culture - Willingness to include sustainability standards - Communication of sustainability goals
Coordinated efforts to align and increase the use of recycled materials in new products - Industry-wide collaborations - Engagement of suppliers - Engagement of business-to-business customers
Consumer appreciation and involvement aimed at increasing the uptake of recycled materials - Engagement of consumers
**Institutional factors** are the policies, rules, regulations, taxes, and infrastructure that directly or indirectly influence the decision-making of companies
Identified research themes:
Efforts for alignment and standardization to increase the uptake of recycled materials - Industry standards for devulcanization - Industry standards (general)
Accountability mechanisms of manufacturers that impact the use of recycled materials - Identified accountability mechanisms - Identified collaborations in industry
Alignment of regulations across sectors and regions that impact the use of recycled materials - Regulations (general) - Regulations across sectors - Regulations across national borders

## References

[1] Winkeman H.A., Busenburg E.B. (1929). Effect of Stearic Acid on Reclaimed Rubber. Ind. Eng. Chem. Ind. Ed..

[2] Markl E., Lackner M. (2020). Devulcanization technologies for recycling of tire-derived rubber: A review. Materials.

[3] Wiśniewska P., Wang S., Formela K. (2022). Waste tire rubber devulcanization technologies: State-of-the-art, limitations and future perspectives. Waste Manag..

[4] Roetman E., Joustra J., Heideman G., Balkenende R. (2024). Does the Rubber Meet the Road? Assessing the Potential of Devulcanization Technologies for the Innovation of Tire Rubber Recycling. Sustainability.

[5] Ghosh R. (2024). Transforming Silica-Silane Reinforced Rubber into a High Quality Devulcanizate.

[6] van Hoek H. (2022). Closing the Loop. Reuse of Devulcanized Rubber in New Tires. Ph.D. Thesis.

[7] Bandyopadhyay S., Bireswar B. (2024). Chapter 1 Fundamentals of rubber compounding. Rubber Products.

[8] De S.K., White J.R. (2001). Rubber Technologist’s Handbook.

[9] Saiwari S., Dierkes W., Letcher T.M., Shulman V.L., Amirkhanian S. (2021). 7—Regeneration and devulcanization. Tire Waste and Recycling.

[10] Shanbag A., Manjare S. (2020). Life cycle assessment of tyre manufacturing process. J. Sustain. Dev. Energy Water Environ. Syst..

[11] Wiśniewska P., Haponiuk J.T., Colom X., Saeb M.R. (2023). Green Approaches in Rubber Recycling Technologies: Present Status and Future Perspective. ACS Sustain. Chem. Eng..

[12] European Commission (2023). Study on the Critical Raw Materials for the EU 2023—Final Report.

[13] Ellen MacArtur Foundation What Is a Circular Economy?. https://www.ellenmacarthurfoundation.org/topics/circular-economy-introduction/overview.

[14] Rodgers B., Waddell W., Mark J.E., Erman B., Roland C.M. (2013). Chapter 9—The Science of Rubber Compounding. The Science and Technology of Rubber.

[15] Sisanth K.S., Thomas M.G., Abraham J., Thomas S., Thomas S., Maria H.J. (2017). 1—General introduction to rubber compounding. Progress in Rubber Nanocomposites.

[16] Lacroix F., Seghar S. (2021). In-Product-Sorting End-of-Life Tires for Effective Recycling by Devulcanization.

[17] Boon Z.H., Teo Y.Y., Ang D.T.-C. (2022). Recent development of biodegradable synthetic rubbers and bio-based rubbers using sustainable materials from biological sources. RSC Adv..

[18] Samsuri A. (2010). Degradation of Natural Rubber and Synthetic Elastomers. Shreir's Corros..

[19] Malaysian Rubber Board (2024). Consumption of Natural and Synthetic Rubber Worldwide from 1990 to H1 2024 (in Million Metric Tons) [Graph]. In Statista. https://www.statista.com/statistics/275399/world-consumption-of-natural-and-synthetic-caoutchouc/.

[20] ETRMA The ETRMA Statistics Report. https://www.etrma.org/wp-content/uploads/2021/12/20211030-Statistics-booklet-2021VF.pdf.

[21] ETRMA (2011). European Tyre & Rubber Industry Statistics Report.

[22] Jaeger B., Upadhyay A. (2020). Understanding barriers to circular economy: Cases from the manufacturing industry. J. Enterp. Inf. Manag..

[23] Kirchherr J., Piscicelli L., Bour R., Kostense-Smit E., Muller J., Huibrechtse-Truijens A., Hekkert M. (2018). Barriers to the Circular Economy: Evidence From the European Union (EU). Ecol. Econ..

[24] Campbell S., Greenwood M., Prior S., Shearer T., Walkem K., Young S., Bywaters D., Walker K. (2020). Purposive sampling: Complex or simple? Research case examples. J. Res. Nurs..

[25] Chaminade C., Edquist C. (2006). From theory to practice: The use of the systems of innovation approach in innovation policy. Innovation, Science, and Institutional Change: A Research Handbook.

[26] Knott E., Rao A.H., Summers K., Teeger C. (2022). Interviews in the social sciences. Nat. Rev. Methods Primers.

[27] Tyrepress The World’s Leading Tyre Makers of 2023. https://www.tyrepress.com/wp-content/uploads/2023/06/Leading-Companies-2023.pdf.

[28] Grafström J., Aasma S. (2021). Breaking circular economy barriers. J. Clean. Prod..

[29] Paletta A., Leal Filho W., Balogun A.-L., Foschi E., Bonoli A. (2019). Barriers and challenges to plastics valorisation in the context of a circular economy: Case studies from Italy. J. Clean. Prod..

[30] de Jesus A., Mendonça S. (2018). Lost in Transition? Drivers and Barriers in the Eco-innovation Road to the Circular Economy. Ecol. Econ..

[31] van der Vegt M., Velzing E.-J., Rietbergen M., Hunt R. (2022). Understanding Business Requirements for Increasing the Uptake of Recycled Plastic: A Value Chain Perspective. Recycling.

[32] Baldassarre B., Maury T., Mathieux F., Garbarino E., Antonopoulos I., Sala S. (2022). Drivers and Barriers to the Circular Economy Transition: The Case of Recycled Plastics in the Automotive Sector in the European Union. Procedia CIRP.

[33] Pinheiro M.F., Ferreira L.M.D.F., Azevedo S.G., Magalhães V.S.M. (2024). Barriers to the Circular Economy in the Plastics Industry: A Systematic Literature Review. Proceedings of the Flexible Automation and Intelligent Manufacturing: Establishing Bridges for More Sustainable Manufacturing Systems.

[34] Shen L., Worrell E., Meskers C., Worrell E., Reuter M.A. (2024). Chapter 31—Plastic recycling. Handbook of Recycling.

[35] Bening C.R., Pruess J.T., Blum N.U. (2021). Towards a circular plastics economy: Interacting barriers and contested solutions for flexible packaging recycling. J. Clean. Prod..

[36] Alassali A., Picuno C., Chong Z.K., Guo J., Maletz R., Kuchta K. (2021). Towards Higher Quality of Recycled Plastics: Limitations from the Material’s Perspective. Sustainability.

[37] Milios L., Holm Christensen L., McKinnon D., Christensen C., Rasch M.K., Hallstrøm Eriksen M. (2018). Plastic recycling in the Nordics: A value chain market analysis. Waste Manag..

[38] McKinnon D., Bakas I., Herczeg M., Vea E.B., Busch N., Christensen L.H., Christensen C., Damgaard C.K., Milios L., Punkkinen H. (2018). Plastic Waste Markets: Overcoming Barriers to Better Resource Utilisation.

[39] Agarwal S., Tyagi M., Garg R.K. (2023). Conception of circular economy obstacles in context of supply chain: A case of rubber industry. Int. J. Product. Perform. Manag..

[40] Schultz F.C., Valentinov V., Kirchherr J., Reinhardt R.J., Pies I. (2024). Stakeholder governance to facilitate collaboration for a systemic circular economy transition: A qualitative study in the European chemicals and plastics industry. Bus. Strategy Environ..

[41] Saldana J. (2015). The Coding Manual for Qualitative Researchers.

[42] Cardona-Uribe N., Betancur M., Martínez J.D. (2021). Towards the chemical upgrading of the recovered carbon black derived from pyrolysis of end-of-life tires. Sustain. Mater. Technol..

[43] Bowles A.J., Fowler G.D. (2022). Assessing the impacts of feedstock and process control on pyrolysis outputs for tyre recycling. Resour. Conserv. Recycl..

[44] Costa S.M.R., Fowler D., Carreira G.A., Portugal I., Silva C.M. (2022). Production and Upgrading of Recovered Carbon Black from the Pyrolysis of End-of-Life Tires. Materials.

[45] Simon P., Plessis S., Ghanimi A. (2024). Evaluating the Path to a Sustainable Tire Industry: Unlocking the Potential of Recovered Carbon Black. https://www.emerton.co/news/evaluating-the-path-to-a-sustainable-tire-industry-unlocking-the-potential-of-recovered-carbon-black.

[46] Schyns Z.O.G., Shaver M.P. (2021). Mechanical Recycling of Packaging Plastics: A Review. Macromol. Rapid Commun..

[47] Plastics Europe Annual Production of Plastics Worldwide from 1950 to 2022 (in Million Metric Tons) [Graph] in Statista. https://www.statista.com/statistics/282732/global-production-of-plastics-since-1950/.

[48] Dwivedi C., Manjare S., Rajan S.K. (2020). Recycling of waste tire by pyrolysis to recover carbon black: Alternative & environment-friendly reinforcing filler for natural rubber compounds. Compos. Part B Eng..

[49] Semperit (2024). Sustainability Report 2023.

[50] Demets R., Van Kets K., Huysveld S., Dewulf J., De Meester S., Ragaert K. (2021). Addressing the complex challenge of understanding and quantifying substitutability for recycled plastics. Resour. Conserv. Recycl..

[51] Auer M., Schmidt J., Diemert J., Gerhardt G., Renz M., Galler V., Woidasky J. (2023). Quality Aspects in the Compounding of Plastic Recyclate. Recycling.

[52] Jager R., Vlugter J., Gort I. (2023). Gevolgen National Norm Circulaire Plastics. Verkenning naar de gevolgen van de Verplichting Voor een Minimumaandeel Recyclaat en/of Biogebaseerd Kunststof en Rubber Voor Nederlandse Converters.

[53] Kratina O., Stoček R., Voldánová J. (2022). How the rubber compounds of different tire’s components heat up?. Constitutive Models for Rubber XII.

[54] Wiesinger H., Wang Z., Hellweg S. (2021). Deep Dive into Plastic Monomers, Additives, and Processing Aids. Environ. Sci. Technol..

[55] Anjum A. (2021). Recovered Carbon Black from Waste Tire Pyrolysis: Characteristics, Performance, and Valorisation.

[56] Gall M., Freudenthaler P.J., Fischer J., Lang R.W. (2021). Characterization of Composition and Structure–Property Relationships of Commercial Post-Consumer Polyethylene and Polypropylene Recyclates. Polymers.

[57] Hahladakis J.N., Velis C.A., Weber R., Iacovidou E., Purnell P. (2018). An overview of chemical additives present in plastics: Migration, release, fate and environmental impact during their use, disposal and recycling. J. Hazard. Mater..

[58] Norris C.J., Cerdán A.L., ter Haar P. (2023). Understanding Recovered Carbon Black. Rubber Chem. Technol..

[59] Schlossnikl J., Pinter E., Jones M.P., Koch T., Archodoulaki V.-M. (2024). Unexpected obstacles in mechanical recycling of polypropylene labels: Are ambitious recycling targets achievable?. Resour. Conserv. Recycl..

[60] Chea J.D., Yenkie K.M., Stanzione J.F., Ruiz-Mercado G.J. (2023). A generic scenario analysis of end-of-life plastic management: Chemical additives. J. Hazard. Mater..

[61] Ghosh A. (2021). Performance modifying techniques for recycled thermoplastics. Resour. Conserv. Recycl..

[62] Dorigato A. (2021). Recycling of polymer blends. Adv. Ind. Eng. Polym. Res..

[63] Orzan E., Janewithayapun R., Gutkin R., Lo Re G., Kallio K. (2021). Thermo-mechanical variability of post-industrial and post-consumer recyclate PC-ABS. Polym. Test..

[64] Moulin L., Da Silva S., Bounaceur A., Herblot M., Soudais Y. (2017). Assessment of recovered carbon black obtained by waste tires steam water thermolysis: An industrial application. Waste Biomass Valorization.

[65] Kong D., Wang S., Shan R., Gu J., Yuan H., Chen Y. (2024). Characteristics and chemical treatment of carbon black from waste tires pyrolysis. J. Anal. Appl. Pyrolysis.

[66] Cabot Corporation (2024). Industry User Guide: Reclaimed Carbon Blending Process.

[67] Lubongo C., Bin Daej M.A.A., Alexandridis P. (2024). Recent Developments in Technology for Sorting Plastic for Recycling: The Emergence of Artificial Intelligence and the Rise of the Robots. Recycling.

[68] Yang J., Xu Y.-P., Chen P., Li J.-Y., Liu D., Chu X.-L. (2023). Combining spectroscopy and machine learning for rapid identification of plastic waste: Recent developments and future prospects. J. Clean. Prod..

[69] Neo E.R.K., Yeo Z., Low J.S.C., Goodship V., Debattista K. (2022). A review on chemometric techniques with infrared, Raman and laser-induced breakdown spectroscopy for sorting plastic waste in the recycling industry. Resour. Conserv. Recycl..

[70] Nyuk Khui P.L., Rahman M.R., Matin M.M., Bin Bakri M.K., Rahman M.R., Bin Bakri M.K. (2022). 7—Recycled rubber waste plastic and its composites. Recycled Plastic Biocomposites.

[71] Okoye C.O., Jones I., Zhu M., Zhang Z., Zhang D. (2021). Manufacturing of carbon black from spent tyre pyrolysis oil—A literature review. J. Clean. Prod..

[72] Martınez J.D., Gisele Jung C., Bouysset J.P. (2021). Recovered carbon black: Potential markets. Tire Waste Recycl..

[73] Faraca G., Martinez-Sanchez V., Astrup T.F. (2019). Environmental life cycle cost assessment: Recycling of hard plastic waste collected at Danish recycling centres. Resour. Conserv. Recycl..

[74] Bridgestone, Michelin (2023). Whitepaper Recovered Carbon Black Guidelines. https://cxf-prod.azureedge.net/b2b-experience-production/attachments/clp2vx72q0l6y01odzd738y3d-rcb-white-paper.pdf.

[75] Kahlert S., Bening C.R. (2022). Why pledges alone will not get plastics recycled: Comparing recyclate production and anticipated demand. Resour. Conserv. Recycl..

[76] Calzolari T., Genovese A., Brint A. (2021). The adoption of circular economy practices in supply chains—An assessment of European Multi-National Enterprises. J. Clean. Prod..

[77] Donnet J.-B. (2018). Carbon Black: Science and Technology.

[78] Kumar A., Pinto P., Hawaldar I.T., Spulbar C.M., Birau F.R. (2021). Crude oil futures to manage the price risk of natural rubber: Empirical evidence from India. Agric. Econ..

[79] Taylor B. BIR Autumn 2024: A Year of Setbacks. Profits Have Been Hard to Come by for Plastics Recyclers and Reprocessors in 2024, with Europe’s Dismal Landscape a Particular Disappointment. https://www.recyclingtoday.com/news/plastic-bir-recycling-meeting-challenges-pricing-mandates-unep-singapore-2024/.

[80] European Union (2023). Regulation (EU) 2023/1805 of the European Parliament and of the Council of 13 September 2023 on the Use of Renewable and Low-Carbon Fuels in Maritime Transport, and Amending Directive 2009/16/EC.

[81] Chapman A., Sen K.K., Mochida T., Yoshimoto Y., Kishimoto K. (2024). Overcoming barriers to proactive plastic recycling toward a sustainable future. Environ. Chall..

[82] European Plastics Converters Sustainability/Circular Economy. https://www.plasticsconverters.eu/.

[83] Plastics Europe Sustainability. https://plasticseurope.org/sustainability/.

[84] Tan J., Tan F.J., Ramakrishna S. (2022). Transitioning to a circular economy: A systematic review of its drivers and barriers. Sustainability.

[85] Alliance to End All Plastic Waste Advancing a Circular Economy for Plastic. https://www.endplasticwaste.org/.

[86] Fan Y., Fowler G.D., Zhao M. (2020). The past, present and future of carbon black as a rubber reinforcing filler—A review. J. Clean. Prod..

[87] Cecon V.S., Da Silva P.F., Curtzwiler G.W., Vorst K.L. (2021). The challenges in recycling post-consumer polyolefins for food contact applications: A review. Resour. Conserv. Recycl..

[88] Vilaplana F., Karlsson S. (2008). Quality Concepts for the Improved Use of Recycled Polymeric Materials: A Review. Macromol. Mater. Eng..

[89] Pfaendner R. (2022). Restabilization—30 years of research for quality improvement of recycled plastics review. Polym. Degrad. Stab..

[90] Marcinowski M. Committee D36 on Recovered Carbon Black (rCB). https://www.astm.org/membership-participation/technical-committees/committee-d36.

[91] ASTM (2024). Resolve 2023 Annual Report.

[92] De Tandt E., Demuytere C., Van Asbroeck E., Moerman H., Mys N., Vyncke G., Delva L., Vermeulen A., Ragaert P., De Meester S. (2021). A recycler’s perspective on the implications of REACH and food contact material (FCM) regulations for the mechanical recycling of FCM plastics. Waste Manag..

[93] Waszczyłko-Miłkowska B., Bernat K., Szczepański K. (2024). Assessment of the Quantities of Non-Targeted Materials (Impurities) in Recycled Plastic Packaging Waste to Comply with EU Regulations and Sustainable Waste Management. Sustainability.

[94] European Union (2023). Commission Notice. Guidance Document for Regulation (EU) 2023/1115 on Deforestation-Free Products (C/2024/6789).

[95] Manžuch Z., Akelytė R., Camboni M., Carlander D. (2021). Chemical Recycling of Polymeric Materials from Waste in the Circular Economy. https://www.actu-environnement.com/media/pdf/news-38548-rapport-echa-recyclage-chimique.pdf.

[96] de Römph T.J., Van Calster G. (2018). REACH in a circular economy: The obstacles for plastics recyclers and regulators. Rev. Eur. Comp. Int. Environ. Law.

[97] European Union (2024). Regulation (EU) 2024/1157 of the European Parliament and of the Council of 11 April 2024 on Shipments of Waste, Amending Regulations (EU) No 1257/2013 and (EU) 2020/1056 and Repealing Regulation (EC) No 1013/2006.

[98] European Commission Guidance on the Interpretation of Key Provisions of Directive 2008/98/EC on Waste. https://ec.europa.eu/environment/pdf/waste/framework/guidance_doc.pdf.

[99] Kara S., Hauschild M., Sutherland J., McAloone T. (2022). Closed-loop systems to circular economy: A pathway to environmental sustainability?. CIRP Ann..

[100] Bakker C., Balkenende R., Poppelaars F. (2018). Design for product integrity in a circular economy. Designing for the Circular Economy.

[101] PureCycle Multiple Compounds Successfully Created Using PureCycle Resin. https://www.purecycle.com/blog/multiple-compounds-successfully-created-using-purecycle-resin?utm_source=chatgpt.com.

[102] Uekert T., Singh A., DesVeaux J.S., Ghosh T., Bhatt A., Yadav G., Afzal S., Walzberg J., Knauer K.M., Nicholson S.R. (2023). Technical, Economic, and Environmental Comparison of Closed-Loop Recycling Technologies for Common Plastics. ACS Sustain. Chem. Eng..

[103] European Commission (2020). A New Circular Economy Action Plan for a Cleaner and More Competitive Europe.

[104] Maitre-Ekern E. (2021). Re-thinking producer responsibility for a sustainable circular economy from extended producer responsibility to pre-market producer responsibility. J. Clean. Prod..

[105] Tumu K., Vorst K., Curtzwiler G. (2023). Global plastic waste recycling and extended producer responsibility laws. J. Environ. Manag..

[106] Winternitz K., Heggie M., Baird J. (2019). Extended producer responsibility for waste tyres in the EU: Lessons learnt from three case studies—Belgium, Italy and the Netherlands. Waste Manag..

[107] Campbell-Johnston K., Calisto Friant M., Thapa K., Lakerveld D., Vermeulen W.J.V. (2020). How circular is your tyre: Experiences with extended producer responsibility from a circular economy perspective. J. Clean. Prod..

[108] Joseph A.M., George B., Madhusoodanan K., Alex R. (2016). The current status of sulphur vulcanization and devulcanization chemistry: Devulcanization. Rubber Sci..

[109] Bockstal L., Berchem T., Schmetz Q., Richel A. (2019). Devulcanisation and reclaiming of tires and rubber by physical and chemical processes: A review. J. Clean. Prod..

[110] Saputra R., Walvekar R., Khalid M., Mubarak N.M., Sillanpää M. (2021). Current progress in waste tire rubber devulcanization. Chemosphere.

[111] Zhang L., Wang H., Zhang C., Wang S. (2023). Laboratory testing and field application of devulcanized rubber/SBS composite modified asphalt. Case Stud. Constr. Mater..

[112] Akhtar A.Y., Tsang H.-H. (2024). Dynamic leaching assessment of recycled polyurethane-coated tire rubber for sustainable engineering applications. Chem. Eng. J..

[113] Lapkovskis V., Mironovs V., Kasperovich A., Myadelets V., Goljandin D. (2020). Crumb Rubber as a Secondary Raw Material from Waste Rubber: A Short Review of End-Of-Life Mechanical Processing Methods. Recycling.

[114] Lazorenko G., Kasprzhitskii A., Mischinenko V. (2021). Rubberized geopolymer composites: Effect of filler surface treatment. J. Environ. Chem. Eng..

[115] Faussone G.C., Cecchi T. (2022). Chemical Recycling of Plastic Marine Litter: First Analytical Characterization of The Pyrolysis Oil and of Its Fractions and Comparison with a Commercial Marine Gasoil. Sustainability.

[116] Zaino M.M., Mustaffa N., Hassan A.H., Mustapa A.N., Mamat M.H., Syed-Hassan S.S.A., Rahman S.N.F.S.A., Azli A.A.M., Hamdan K. (2024). A Mini Review on Carbon Black Production as Rubber Reinforcement in Tyre Application. Chem. Eng. Trans..

[117] Czajczyńska D., Krzyżyńska R., Jouhara H., Spencer N. (2017). Use of pyrolytic gas from waste tire as a fuel: A review. Energy.

[118] Gaidhane J., Ullah I., Khalatkar A. (2022). Tyre remanufacturing: A brief review. Mater. Today Proc..

[119] Dabić-Ostojić S., Miljuš M., Bojović N., Glišović N., Milenković M. (2014). Applying a mathematical approach to improve the tire retreading process. Resour. Conserv. Recycl..

[120] Myhre M., MacKillop D.A. (2002). Rubber recycling. Rubber Chem. Technol..

[121] Vasiliu L., Gencel O., Damian I., Harja M. (2023). Capitalization of tires waste as derived fuel for sustainable cement production. Sustain. Energy Technol. Assess..

[122] Ferdous W., Manalo A., Siddique R., Mendis P., Zhuge Y., Wong H.S., Lokuge W., Aravinthan T., Schubel P. (2021). Recycling of landfill wastes (tyres, plastics and glass) in construction—A review on global waste generation, performance, application and future opportunities. Resour. Conserv. Recycl..

[123] Dabrowska D., Rykala W., Nourani V. (2023). Causes, types and consequences of municipal waste landfill fires—Literature review. Sustainability.

